# M2 macrophages induce ovarian cancer cell proliferation via a heparin binding epidermal growth factor/matrix metalloproteinase 9 intercellular feedback loop

**DOI:** 10.18632/oncotarget.13474

**Published:** 2016-11-19

**Authors:** Molly J. Carroll, Arvinder Kapur, Mildred Felder, Manish S. Patankar, Pamela K. Kreeger

**Affiliations:** ^1^ Department of Biomedical Engineering, University of Wisconsin-Madison, WI, USA; ^2^ Department of Obstetrics and Gynecology, University of Wisconsin School of Medicine and Public Health, WI, USA; ^3^ Department of Cell and Regenerative Biology, University of Wisconsin School of Medicine and Public Health, WI, USA

**Keywords:** heparin binding epidermal growth factor, matrix metalloproteinase 9, co-culture, paracrine signaling, bi-directional communication

## Abstract

In ovarian cancer, a high ratio of anti-inflammatory M2 to pro-inflammatory M1 macrophages correlates with poor patient prognosis. The mechanisms driving poor tumor outcome as a result of the presence of M2 macrophages in the tumor microenvironment remain unclear and are challenging to study with current techniques. Therefore, in this study we utilized a micro-culture device previously developed by our lab to model concentrated paracrine signaling in order to address our hypothesis that interactions between M2 macrophages and ovarian cancer cells induce tumor cell proliferation. Using the micro-culture device, we determined that co-culture with M2-differentiated primary macrophages or THP-1 increased OVCA433 proliferation by 10–12%. This effect was eliminated with epidermal growth factor receptor (EGFR) or heparin-bound epidermal growth factor (HB-EGF) neutralizing antibodies and *HBEGF* expression in peripheral blood mononuclear cells from ovarian cancer patients was 9-fold higher than healthy individuals, suggesting a role for HB-EGF in tumor progression. However, addition of HB-EGF at levels secreted by macrophages or macrophage-conditioned media did not induce proliferation to the same extent, indicating a role for other factors in this process. Matrix metalloproteinase-9, MMP-9, which cleaves membrane-bound HB-EGF, was elevated in co-culture and its inhibition decreased proliferation. Utilizing inhibitors and siRNA against *MMP9* in each population, we determined that macrophage-secreted MMP-9 released HB-EGF from macrophages, which increased *MMP9* in OVCA433, resulting in a positive feedback loop to drive HB-EGF release and increase proliferation in co-culture. Identification of multi-cellular interactions such as this may provide insight into how to most effectively control ovarian cancer progression.

## INTRODUCTION

Ovarian cancer is the most lethal gynecological cancer, with 75% of patients presenting with tumors that have already metastasized throughout the peritoneum [1]. The environment of these metastatic tumors is diverse, with multiple cell types that potentially drive tumor progression, which could provide targets to slow or stop metastatic disease. Despite the importance of these interactions, analysis of the mechanisms involved remains challenging due to the complexity of *in vivo* models and limitations of standard *in vitro* setups.

Stromal cells found in the ovarian cancer metastatic microenvironment include fibroblasts, adipocytes, mesothelial cells, and immune cells [2], with macrophages the most abundant immune cell type [3]. Macrophages can be characterized based on their differentiation to either pro-inflammatory (M1) or anti-inflammatory (M2) states [3, 4], and a high ratio of M2 to M1 macrophages has been correlated with poor prognosis in ovarian cancer patients [5]. Despite their potential clinical relevance, the specific mechanisms that account for the impact of M2 macrophages on ovarian cancer progression remain poorly understood. M2 macrophages are an abundant source of cytokines, growth factors, and matrix metalloproteinases (MMPs) [4] that can signal to tumor cells and impact their behavior [6–8]. M2 macrophages have been shown to increase proliferation in other tumor types such as breast cancer [9]. Therefore, we hypothesized that paracrine signaling between M2 macrophages and ovarian cancer cells would increase tumor cell proliferation. To address our hypothesis, we utilized a micro-culture device we recently developed that allows for paracrine signaling between two cell populations [10]. Our data suggests that crosstalk between the two cell types results in a positive feedback loop that drives tumor cell proliferation.

## RESULTS

### M2 MDMs increase OVCA433 proliferation through an EGFR mechanism

**Figure 1:**
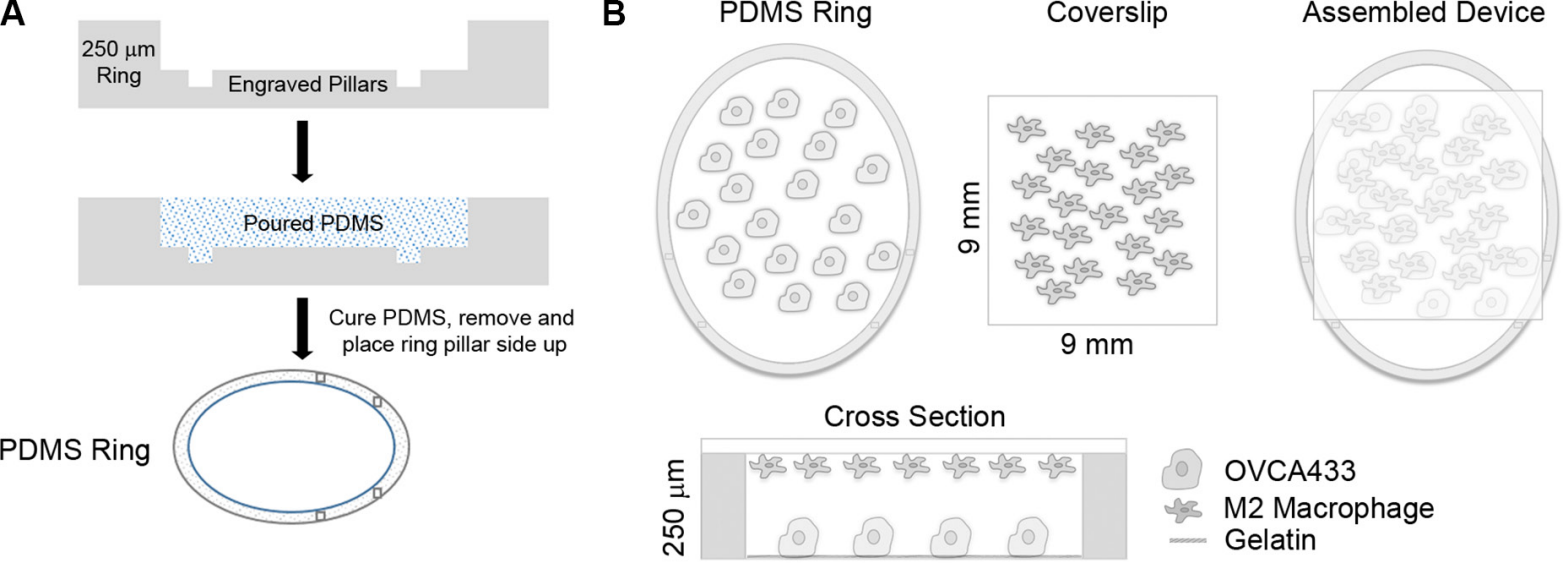
Overview of micro-culture device (**A**) Schematic of PDMS ring construction. (**B**) Schematic of OVCA433 and M2 macrophages in co-culture device.

**Figure 2:**
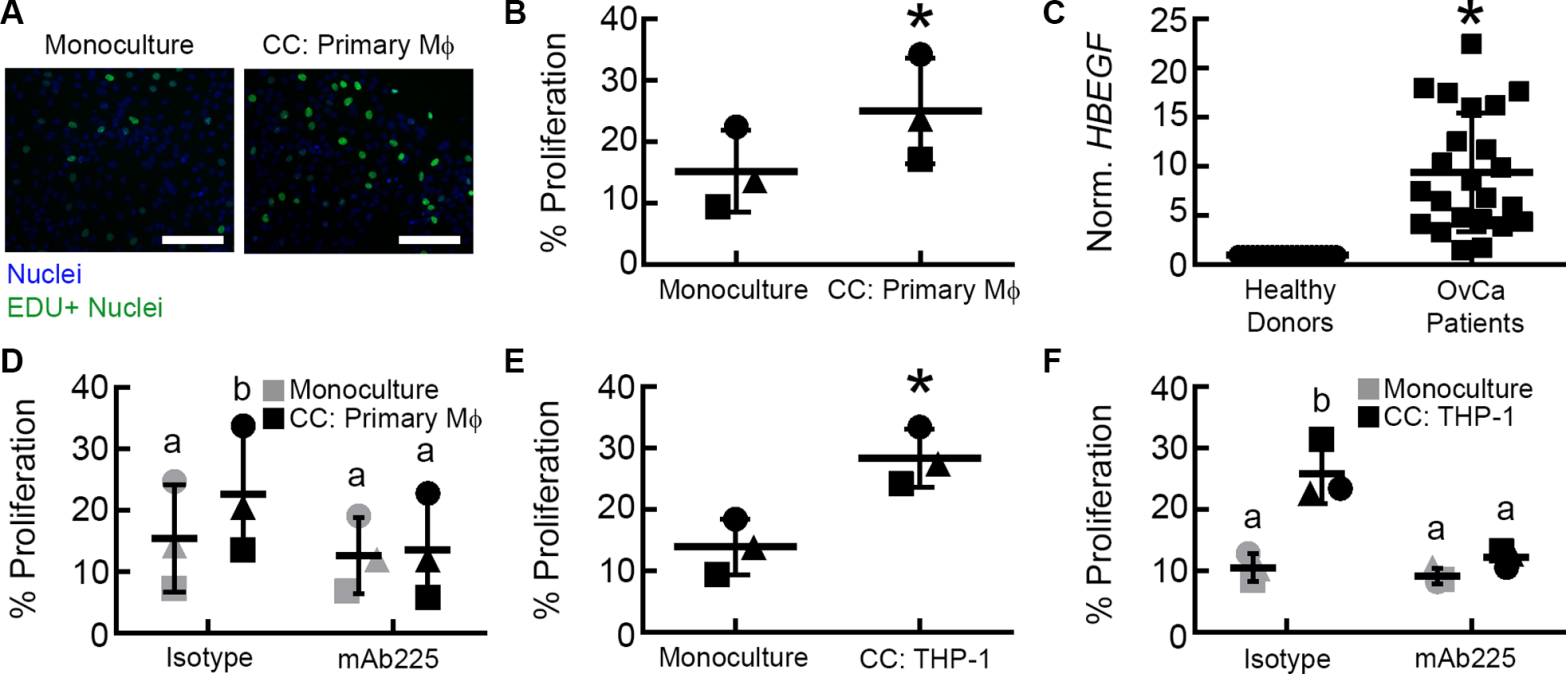
Paracrine signaling between M2 macrophages and OVCA433 increases tumor proliferation via EGFR (**A**) Example of Click iT EdU fluorescent microscopy images from monoculture and co-culture with primary macrophages (CC: Primary Mϕ), scale bar = 100 μm. (**B**) Impact of M2 MDM co-culture (CC: Primary Mϕ) on OVCA433 proliferation. Shown are results from three unique donors, different symbols indicate each donor, *p < 0.05 compared to monoculture. (**C**) *HBEGF* expression in PBMCs from a separate cohort of 23 ovarian cancer patients relative to 21 healthy donors, *p < 0.05 compared to healthy donors. (**D**) Impact of mAb225 (10 μg/mL) on OVCA433 proliferation in monoculture and co-culture with three unique donors (CC: Primary Mϕ), different letters indicate that two conditions are significantly different, *p* < 0.05. (**E**) Impact of M2 THP-1 co-culture (CC: THP-1) on OVCA433 proliferation. Shown are results from three biological replicates, *p < 0.05 compared to monoculture. (**F**) Impact of mAb225 (10 μg/mL) on OVCA433 proliferation in monoculture and co-culture (CC: THP-1) from three biological replicates, different letters indicate that two conditions are significantly different, *p* < 0.05.

Interactions between tumor-associated (M2) macrophages and tumor cells have been suggested to play an important role in ovarian cancer [3], but remain difficult to study with existing experimental models. We recently developed a micro-device that allows for two cell types to be cultured in parallel, allowing for the exchange of soluble factors [10]. The small volume of this system (40 μL) maintains these secreted factors at high concentrations relative to standard culture setups (*e.g*., transwells). Using this system (Figure 1), we examined the paracrine interactions between primary M2 MDMs derived from healthy female donors and OVCA433, an ovarian cancer line with a *TP53* mutation [11]. The M2 phenotype of donor MDMs was confirmed by immunofluorescence for CD68 and CD206 expression (Supplementary Figure S1). After 48 hours of co-culture with M2 MDMs, OVCA433 had significantly increased proliferation compared to monoculture controls (Figure 2A, 2B). We hypothesized that ligands secreted by M2 macrophages were responsible for the increased OVCA433 proliferation in co-culture. EGFR ligands, including EGF, TGFα, and HB-EGF, have all been suggested to enhance ovarian cancer progression [12–14] and increase tumor cell proliferation [7, 15–17]. Of the EGFR ligands, macrophages have been previously reported to secrete HB-EGF, but not TGFα or EGF [18, 19]. qRT-PCR analysis confirmed the pattern of *HBEGF*-positive, *TGFA/EGF* negative in our M2 MDMs (Supplementary Table S2). Monocytes are the primary immune cell in PBMCs that secrete HB-EGF [20]; therefore, we compared expression of *HBEGF* in PBMCs of healthy donors and ovarian cancer patients to determine if HB-EGF may play a role in ovarian cancer. qRT-PCR demonstrated that *HBEGF* expression in PBMCs from ovarian cancer patients was 9-fold higher than in healthy donors (Figure 2C), and flow cytometry confirmed that the monocyte population was positive for HB-EGF (Supplementary Figure S2).

To determine if EGFR ligands were responsible for the observed effect of co-culture on proliferation, M2 MDM-OVCA433 co-cultures were treated with mAb225, a monoclonal antibody that blocks ligand binding to EGFR [21]. mAb225 had no impact on OVCA433 proliferation in the monoculture condition, indicating minimal autocrine EGFR activity. In contrast, blocking EGFR inhibited proliferation in M2 MDM co-culture (Figure 2D), suggesting that EGFR ligands secreted by M2 MDMs were responsible for the increase in OVCA433 proliferation. In order to examine in detail the mechanism responsible for the observed effect of macrophage co-culture, we utilized the THP-1 monocytic-like cell line. This monocytic-like model cell line has been used previously to investigate the functions of macrophages [22]. We first confirmed that, similar to primary M2 MDMs (Figure 2B), M2 THP-1 induced OVCA433 proliferation in co-culture (Figure 2E). Additionally, as seen with primary MDMs (Figure 2D), blocking EGFR with mAb225 reduced OVCA433 proliferation in M2 THP-1 co-culture to the level of monoculture conditions (Figure 2F).

### Paracrine HB-EGF from M2 THP-1 increases OVCA433 proliferation in co-culture

**Figure 3:**
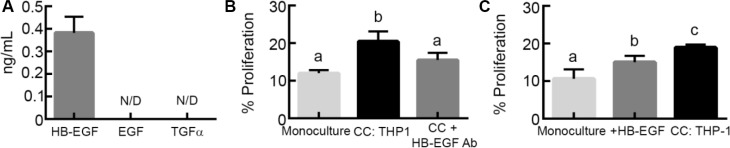
M2 macrophage-secreted HB-EGF drives tumor cell proliferation in co-culture (**A**) Levels of EGFR ligands secreted by M2 THP-1, N/D indicates not detected. (**B**) Comparison of proliferation in OVCA433 monoculture co-culture with THP-1 (CC: THP-1), and co-culture with HB-EGF neutralizing antibody (10 μg/mL; CC + HB-EGF Ab) after 48 hours, different letters indicate that two conditions are significantly different, *p* < 0.05. (**C**) Impact of treatment with HB-EGF (400 pg/mL; + HB-EGF) on OVCA433 proliferation in monoculture compared to M2 THP-1 co-culture (CC: THP-1) after 48 hours, different letters indicate that two conditions are significantly different, *p* < 0.05.

To our knowledge, while EGFR ligands have been characterized for M2 MDMs, the levels produced by M2 THP-1 have not been characterized. Therefore, we next quantified the levels of EGFR ligands secreted by M2 THP-1 by ELISA and determined that, like M2 MDMs [18, 19], M2 THP-1 secreted HB-EGF, but not EGF or TGFα (Figure 3A). To confirm that HB-EGF secreted by M2 THP-1 was responsible for the macrophage-induced proliferation, OVCA433 in monoculture and co-culture with M2 THP-1 were treated with an HB-EGF neutralizing antibody. As seen with mAb225, neutralizing HB-EGF did not impact baseline proliferation, but significantly inhibited M2 THP-1 induced proliferation (Figure 3B). To further confirm that HB-EGF was responsible for the phenotypic changes in M2 THP-1 co-cultures, monoculture and M2 THP-1 co-cultures were compared to OVCA433 treated with 400 pg/mL HB-EGF, a concentration that matched the level produced by M2 THP-1 in monoculture (Figure 3A) and that is similar to levels detected in the ascites fluid of patients [23]. While OVCA433 treated with HB-EGF had significantly increased proliferation compared to monocultures, this level was still significantly less than in M2 THP-1 co-cultures (Figure 3C). These results suggest that while HB-EGF was necessary to induce proliferation, other ligands secreted by M2 THP-1 may contribute to the observed increase in proliferation with M2 THP-1 co-culture.

### Matrix metalloproteinases increase OVCA433 proliferation in co-culture

**Figure 4:**
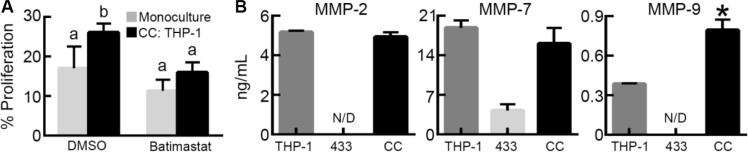
M2 macrophage-secreted matrix metalloproteinases contribute to tumor cell proliferation (**A**) Impact of batimastat (10 μM) on OVCA433 proliferation in co-culture with M2 THP-1 (CC: THP-1) after 48 hours, different letters indicate that two conditions are significantly different, *p* < 0.05. (**B**) Concentration of MMP-2, MMP-7, and MMP-9 in M2 THP-1 monoculture (THP-1), OVCA433 monoculture (433), and OVCA433 co-culture with M2 THP-1 (CC), *p < 0.05 compared to M2 THP-1 monoculture, N/D indicates not detected.

HB-EGF is initially tethered to the cell membrane, but can be processed to a soluble form by MMPs to participate in paracrine signaling [24] and M2 macrophages are a source of MMPs in the tumor microenvironment [4]. Therefore, we sought to determine if MMPs played a role in the increased proliferation observed in co-culture. To inhibit MMP activity, we treated cells with batimastat, a broad MMP inhibitor that has specificity towards MMP-1, -2, -3, -7, and -9 [25]. When OVCA433 in monoculture were treated with batimastat (10 μM), no changes in proliferation were observed relative to vehicle (Figure 4A). However, when M2 THP-1 were co-cultured with OVCA433 and treated with batimastat, tumor cell proliferation was significantly reduced in comparison to the vehicle treated co-cultures (Figure 4A), suggesting that MMPs secreted by macrophages played a role in the observed increase in OVCA433 proliferation. The MMPs that are inhibited by batimastat that have also been reported to cleave HB-EGF from the surface of cells are MMP-2, -7, and -9 [19, 26, 27]. Analysis of media collected from monocultures indicated that both OVCA433 and M2 THP-1 secreted MMP-7, while MMP-2 and -9 were secreted by M2 THP-1 only (Figure 4B). Intriguingly, the level of MMP-9 in co-culture was significantly increased compared to M2 THP-1 in monoculture, suggesting a potential role for MMP-9 in the observed effects of co-culture.

### MMP-9 induces proliferation of OVCA433 through an indirect mechanism

**Figure 5:**
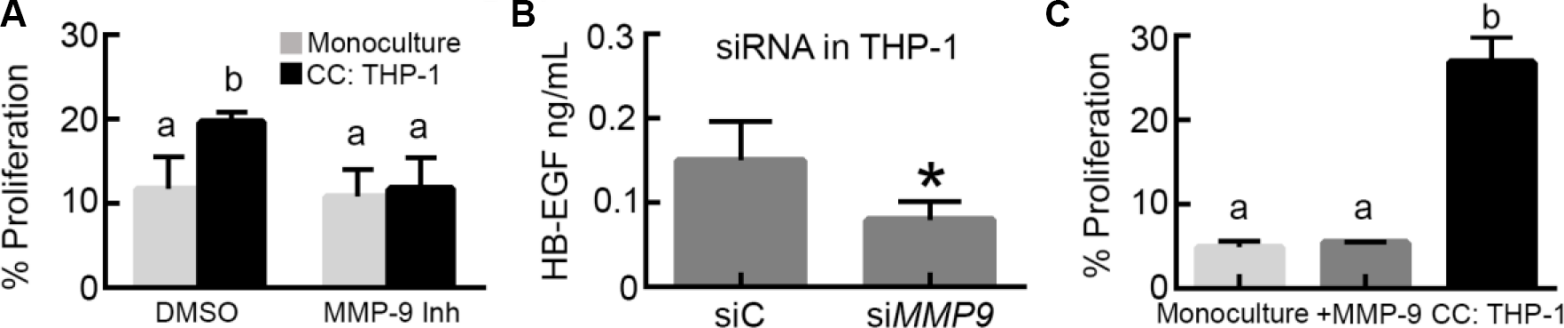
MMP-9 impacts tumor cell proliferation via control of HB-EGF secretion (**A**) Impact of MMP-9 inhibitor (5 nM) on OVCA433 proliferation in co-culture with M2 THP-1 (CC: THP-1) after 48 hours, different letters indicate that two conditions are significantly different, *p* < 0.05. (**B**) Impact of *MMP9* siRNA knockdown in M2 THP-1 (si*MMP9*) on HB-EGF release, *p < 0.05 compared to siRNA control transfected M2 THP-1 (siC). (**C**) Impact of 800 pg/mL of active MMP-9 on OVCA433 proliferation (+ MMP-9) compared to monoculture and THP-1 co-culture (CC: THP-1) after 48 hours, different letters indicate that two conditions are significantly different, *p* < 0.05.

We hypothesized that MMP-9, present in co-culture at elevated levels compared to OVCA433 monoculture, may be involved in the mechanism of increased OVCA433 proliferation. To test this hypothesis, we used a specific MMP-9 inhibitor to determine its effect on proliferation in co-culture. MMP-9 inhibition had no effect on OVCA433 proliferation in monoculture; in contrast, MMP-9 inhibition significantly reduced proliferation in M2 THP-1 co-culture to levels comparable to monoculture (Figure 5A), suggesting that MMP-9 was necessary for the induction of proliferation. While MMP-9 has been suggested to cleave HB-EGF and increase its bioavailability [19, 27, 28], this mechanism has not been confirmed in macrophages. Therefore, to verify MMP-9 induced shedding of HB-EGF in THP-1, media was collected from THP-1 transfected with siRNA against *MMP9* or nonspecific siRNA (Supplementary Figure S3A, S3B) and analyzed by HB-EGF ELISA. Results demonstrated that knockdown of MMP-9 in THP-1 cells decreased the concentration of HB-EGF in THP-1 conditioned media (Figure 5B). Although MMP-9 regulated HB-EGF release from THP-1, it is possible that the observed dependency of tumor cell proliferation on MMP-9 was the result of MMP-9 acting directly on the tumor cells versus the macrophages. To test this possibility, OVCA433 in monoculture were treated with concentrations of active MMP-9 comparable to the level secreted by THP-1 (800 pg/mL, Figure 4B). Direct addition of active MMP-9 had no effect on OVCA433 proliferation (Figure 5C), suggesting that MMP-9 did not directly impact tumor cells, but instead had an indirect role in co-culture induced proliferation.

### HB-EGF and MMP-9 feedback loop drives increase in OVCA433 proliferation in co-culture

**Figure 6:**
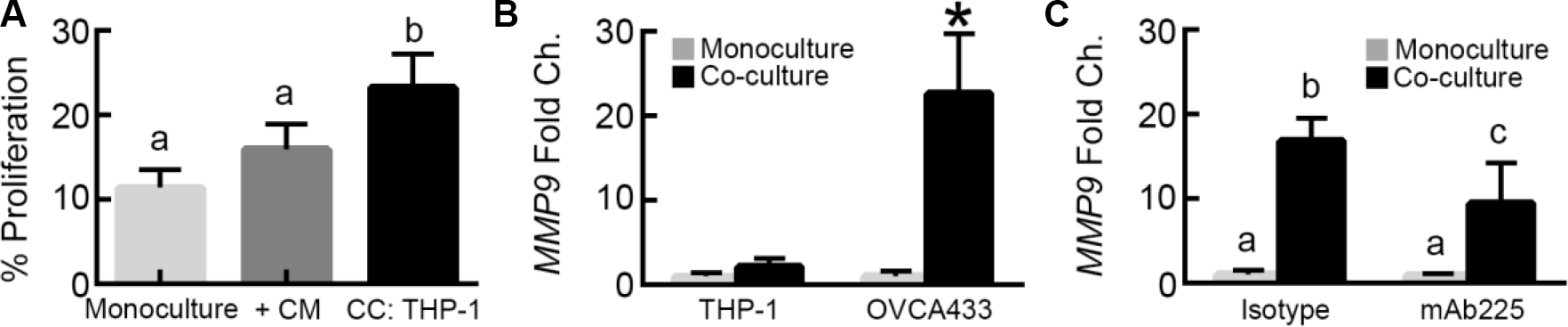
MMP-9 in tumor cells is up-regulated via EGFR signaling (**A**) Impact of THP-1 conditioned media on OVCA433 proliferation (+CM) compared to monoculture and THP-1 co-culture (CC: THP-1) after 48 hours, different letters indicate that two conditions are significantly different, *p* < 0.05. (**B**) *MMP9* expression in M2 THP-1 and OVCA433 mono/co-cultures, *p < 0.05 compared to monoculture for each cell type. (**C**) Impact of 10 μg/mL mAb225 on *MMP9* expression in OVCA433 in monoculture or co-culture with THP-1, different letters indicate that two conditions are significantly different, *p* < 0.05.

Our findings suggested that HB-EGF released by THP-1 macrophages under the control of MMP-9 was necessary, but not sufficient for the observed increase in proliferation with co-culture. To determine whether additional THP-1 secreted factors were also responsible for this effect, OVCA433 monocultures were treated with M2 THP-1 conditioned media. This treatment induced a small, but not significant, increase in proliferation (Figure 6A), suggesting that cross talk between the tumor cells and macrophages that would occur in a dynamic co-culture, but not with conditioned media, may be necessary for the full effect of co-culture on proliferation. We had observed that the MMP-9 concentration increased significantly in co-culture compared to THP-1 monoculture (Figure 4B); therefore, we hypothesized that this increase might drive release of additional HB-EGF. To determine the source of this additional MMP-9, we examined which cell type upregulated *MMP9* expression. We have previously demonstrated that our micro-device design allowed for measurement of RNA without contamination between the two cell types [10]. Using qRT-PCR, it was found that THP-1 *MMP9* expression did not change between monoculture and co-culture; however, OVCA433 *MMP9* expression increased 20-fold in co-culture (Figure 6B). Prior studies demonstrated that OVCA433 treated with high concentrations of EGF (124 ng/mL) induced secretion of MMP-9 [29]. To determine whether OVCA433 *MMP9* upregulation in co-culture was due to EGFR activation, we examined changes in OVCA433 *MMP9* expression when EGFR was inhibited using mAb225 during co-culture. Treatment with mAb225 significantly reduced *MMP9* up-regulation, demonstrating a role for activation of EGFR in this process even at the lower levels of HB-EGF that were observed in co-culture (Figure 6C).

**Figure 7:**
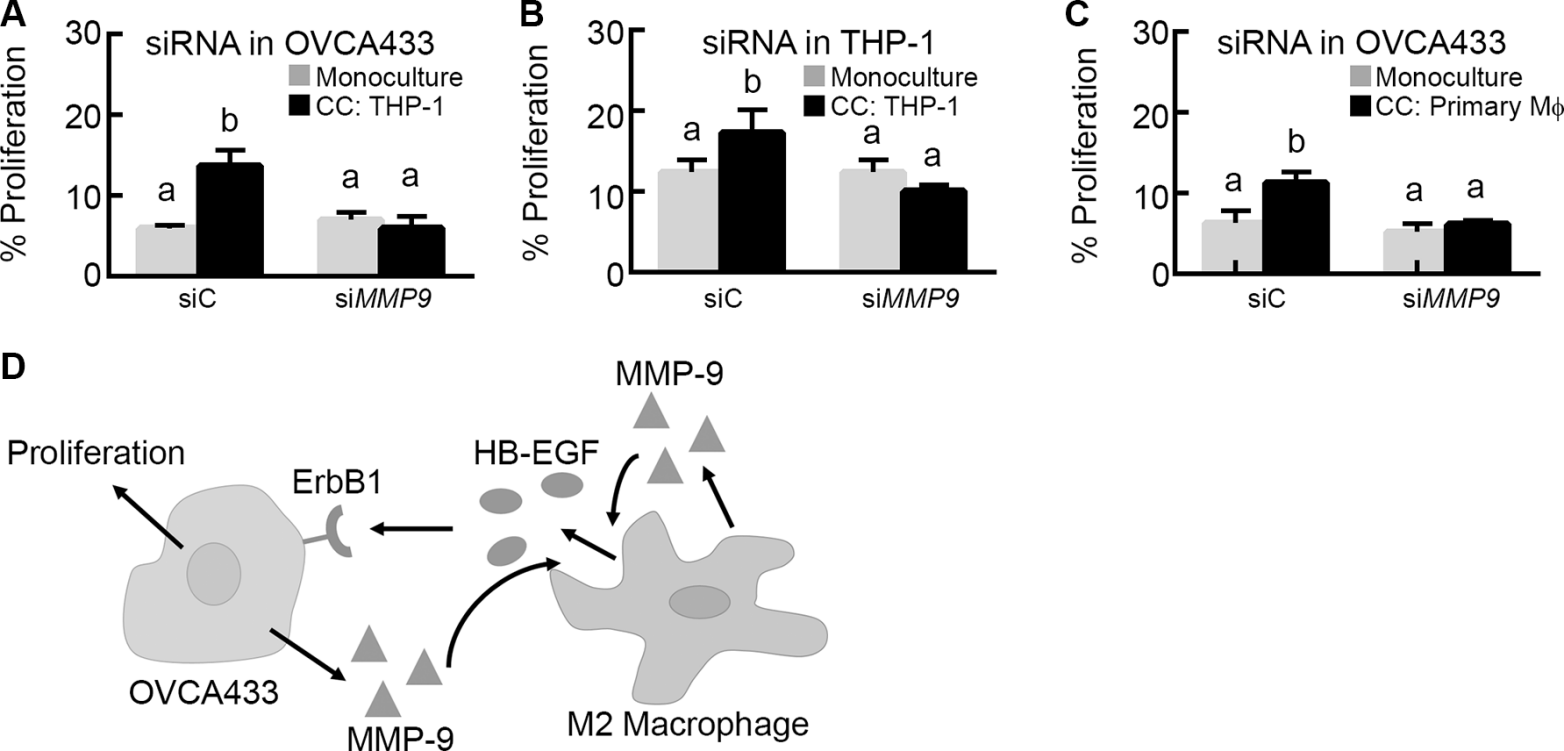
MMP-9 creates a feedback loop with HB-EGF to increase its bioavailability and drive tumor cell proliferation (**A**) Impact of *MMP9* (si*MMP9*) versus control (siC) siRNA knockdown in OVCA433 on OVCA433 proliferation in co-culture with M2 THP-1 (CC: THP-1). (**B**) Impact of *MMP9* (si*MMP9*) versus control (siC) siRNA knockdown in M2 THP-1 on OVCA433 proliferation in co-culture (CC: THP-1). (**C**) Impact of *MMP9* (si*MMP9*) versus control (siC) siRNA knockdown in OVCA433 on OVCA433 proliferation in co-culture with M2 MDMs (CC: Primary Mϕ). Different letters indicate that two conditions are significantly different *p* < 0.05. (**D**) Schematic of proposed HB-EGF feedback loop between tumor cells and M2 macrophages.

Next, we wanted to determine whether the increased MMP-9 produced by OVCA433 in co-culture could be a component in a positive feedback loop acting on HB-EGF release to induce OVCA433 proliferation. Since the MMP-9 inhibitor used previously was targeted towards all secreted MMP-9, we utilized siRNA transfected into each cell type. siRNA knockdown of *MMP9* in OVCA433 significantly reduced OVCA433 proliferation in co-culture (Figure 7A and Supplementary Figure S3C), suggesting that MMP-9 secreted by OVCA433 during co-culture feeds back to the THP-1 to release HB-EGF and drive tumor cell proliferation. When *MMP9* was knocked down in THP-1, co-culture proliferation was significantly reduced (Figure 7B). Combined with our prior observation that HB-EGF release was significantly reduced in THP-1 monocultures transfected with siRNA against *MMP9* (Figure 5B) and that OVCA433 do not produce detectable MMP-9 until after extended co-culture (Figure 4B), this result supports that THP-1 secreted MMP-9 was still crucial for the initiation of the cascade of HB-EGF cleavage, EGFR signaling, MMP-9 production, and ultimately proliferation in OVCA433. To determine whether the same feedback mechanism was present with primary M2 MDMs, OVCA433 siRNA studies were performed with donor M2 MDMs. As with the THP-1 studies, EGFR induced expression of *MMP9* in OVCA433 was necessary for co-culture induced proliferation (Figure 7C). Collectively, these results suggest a novel mechanism in which macrophage-secreted MMP-9 cleaves HB-EGF from the macrophage surface, leading to activated EGFR in OVCA433 and up-regulation of MMP-9, creating a positive feedback loop for HB-EGF bioavailability and increased proliferation in tumor cells (Figure 7D).

## DISCUSSION

Multi-cellular interactions are critical in normal homeostasis and diseases such as cancer. However, studying interactions between two different cell types *in vivo* or *in vitro* is challenging with current techniques. While the use of *in vivo* models inherently incorporates multiple cell types, their low-throughput nature and expense limit in-depth study of mechanisms that drive disease progression. *In vitro*, paracrine interactions are primarily modeled through treatment with exogenous factors, conditioned media experiments, or transwells. Studies with exogenous addition of factors suspected to mediate paracrine interactions are typically done at saturating levels, which are substantially higher than levels observed *in vivo*. In our experiments, we found that addition of exogenous HB-EGF at levels similar to those detected in ascites fluid [23] was insufficient to recapitulate the effect of co-culture on proliferation. Conditioned media experiments only allow for one-way communication to be analyzed. Here, the addition of M2 macrophage conditioned media did not induce significant effects on OVCA433 proliferation due the absence of the bi-directional communication between the tumor cells and M2 macrophages. Transwell cultures, while allowing for bi-directional communication, dilute out key ligands produced by cells due to large required media volumes, and may not accurately model the proposed locally high concentration of ligands between neighboring cells in tumors [30]. In order for ligands to incite cellular signaling and changes in downstream behavior, it has been suggested that a threshold of receptor activation may need to be met [31], which is not possible if the ligands are diluted. In previous studies by our group validating this micro-culture device, it was found that increasing the height of the PDMS ring, and thus the volume within the device, muted the impact of THP-1 on OVCA433 proliferation [10]. Our results here support that the use of *in vitro* systems that enable concentrated, dynamic paracrine interactions can identify mechanisms by which macrophages influence ovarian cancer and may be broadly useful to study other cell-cell interactions.

Using our *in vitro* model, we determined that HB-EGF secreted by M2 macrophages induced increased ovarian cancer cell proliferation. Prior studies examining the effect of autocrine HB-EGF on ovarian cancer cells found that HB-EGF increased adhesion, invasion, and VEGF production *in vitro*, and that the HB-EGF inhibitor CRM197 abrogated metastasis in xenograft models [13], suggesting that therapeutically targeting HB-EGF or its receptor EGFR could slow tumor growth. While the targeted EGFR antibody cetuximab has been tested as a single agent in phase II clinical trials with ovarian cancer patients, its effectiveness was limited [32]. In this trial, patients had recurrent disease and the only histological requirement was EGFR expression in the tumor. In order to better identify patients that would benefit from targeted therapy, it may be necessary to stratify patients more precisely. For example, a clinical trial of cetuximab in colorectal cancer found that patients who expressed wild-type KRAS had increased progression-free survival with cetuximab compared to those with mutated KRAS [33]. In order to stratify patients, one possibility could be to incorporate information on the presence of EGFR ligands such as HB-EGF, which are necessary to activate the receptor [34], or MMPs that regulate ligand bioavailability. Clinical studies have previously shown that ovarian cancer patients have increased concentrations of HB-EGF in their peritoneal fluid and serum compared to patients with benign disease [23]. Our analysis of 23 patients indicated that whole blood PBMCs were a source of HB-EGF in serum and that while these levels were higher than in healthy donors, there was substantial variability that may warrant stratification of patients.

We also determined that the presence of MMP-9 in the tumor/macrophage microenvironment indirectly increased tumor cell proliferation. Studies investigating MMP expression in ovarian cancer patients determined that MMP-9 expression correlated with the advanced stage of the disease [35], and clinical data suggested that elevated levels of pro-MMP-9 are of prognostic value, independent of residual disease presence [36]. In multiple tumor types, MMP-9 drives cell behaviors such as epithelial to mesenchymal transition [29] and invasion [37], primarily through the effects of MMP-9 on degradation of extracellular matrix such as collagen I, II, and IV [38, 39]. Our results showed that inhibition of MMP-9 prevented co-culture induced proliferation; however, addition of activated MMP-9 to tumor cells did not increase proliferation. These findings suggest an indirect role for MMP-9 on tumor cell proliferation, possibly through its action on growth factor availability and fine-tuning of EGFR paracrine signaling in co-culture. This function of MMP-9 as a regulator of paracrine interactions is supported by studies with MMP-9 deficient mice, which had delayed neoplastic progression and reduced proliferation in a model of squamous cell carcinoma [40]. Importantly, the introduction of MMP-9 expressing bone marrow cells induced proliferation and neoplastic progression, indicating a crucial role for immune cells in MMP-9 driven cancer progression.

The combined analysis of HB-EGF and MMP-9 actions indicated the presence of a feedback loop between HB-EGF induced production of MMP-9 in ovarian cancer cells and release of soluble HB-EGF from M2 macrophages that led to co-culture induced proliferation. In our identified feedback loop, macrophage-secreted MMP-9 was responsible for initial cleavage of HB-EGF from the macrophage surface, which in turn increased *MMP9* expression in the tumor cells. The importance of this feedback is apparent from the inability of either exogenous HB-EGF or conditioned media containing all factors secreted by macrophages in monoculture to recapitulate the effect of co-culture. This difference is likely due to internalization and degradation of HB-EGF, which results in its depletion from the system [41]. While the concentration of HB-EGF added exogenously or as conditioned media was comparable to that measured from single timepoint analysis of ascites fluid [23], the combination of the small volume in the device and the low concentration resulted in a low amount of HB-EGF available. Without the presence of macrophages to produce additional growth factor, the HB-EGF would be depleted from the media over time. This phenomena is not always observed when experiments are conducted at high doses or large media volumes that result in an excess of growth factor relative to the cellular receptor number [42]. However, ligand depletion is a key method by which cells are able to ‘shut off’ a stimuli. For example, it has been shown that transforming growth factor beta depletion via cellular internalization was the primary determining factor for downstream signaling kinetics in HaCat epithelial cells and PE25 mink lung epithelial cells [43]. A consequence of ligand depletion during the exogenous HB-EGF treatment is that this translates to a short, transient stimulation of cells which may alter cell response. For example, studies examining p38 dynamics determined that long-term exposure of HeLa cells to interleukin 1 beta was necessary to induce p38 effector genes [44]. In our culture system, as *in vivo*, both cell types interact for extended times, providing a continuous source of secreted ligands.

When *MMP9* was knocked down in OVCA433, extended co-culture was unable to induce the expected increase in proliferation, indicating that the induction of *MMP9* in the tumor cells was critical. In both normal and tumor cells, *MMP-9* has been shown to be secreted in response to EGFR activation via exogenous ligands [29, 37, 45]. Our findings showed that ovarian cancer cells could be induced to secrete MMP-9 in response to EGFR activation by physiological levels of HB-EGF. It should be noted that while EGFR inhibition decreased co-culture induced *MMP9* expression, it did not reduce *MMP9* to monoculture levels. Other ligands secreted by M2 macrophages, such as tranforming growth factor beta [46], have been found to induce *MMP9* expression in MCF10A cells [47], and may also play a role in non-EGFR induced *MMP9* expression in this system. This feedback was found to be essential for the effects of co-culture with both the THP-1 model macrophage line and primary M2 macrophages. Therefore, our data not only suggests a role for HB-EGF in ovarian tumor growth, but also identifies macrophages as a likely source of this growth factor and MMP-9 as a mechanism for controlling HB-EGF bioavailability, providing a clinical rationale for targeting both HB-EGF and MMP-9 in the tumor microenvironment.

## MATERIALS AND METHODS

### Cell lines and reagents

Unless noted otherwise, all reagents were purchased from Sigma-Aldrich (St. Louis, MO). The high grade serous ovarian cancer (HGSOC) cell line OVCA433 was obtained from Dr. R. Bast (MD Anderson Cancer Center; Houston, TX) [48]. The peripheral blood acute monocytic line THP-1 was purchased from ATCC (Manassas, VA). OVCA433 were cultured in 1:1 Medium199:MCDB105 supplemented with 10% fetal bovine serum (FBS, Invitrogen; Carlsbad, CA) and 1% penicillin-streptomycin (Invitrogen). OVCA433 was authenticated by human short tandem repeat (STR) analysis at the Translational Research Initiatives in Pathology (TRIP) lab at the University of Wisconsin–Madison. THP-1 were cultured in RPMI (CellGro; Manassas, VA) supplemented with 10% FBS, 1% penicillin-streptomycin, and 50 μM 2-mercaptoethanol.

### Device fabrication

Devices consisted of two parallel culture surfaces (a well within a 24-well tissue culture plate and a glass coverslip) separated by a polydimethylsiloxane (PDMS) ring cast from a negative mold (Figure 1A, [10]). Using an Epilog Mini18 40 Watt Laser Cutter (Epilog Laser Golden, CO), negatives of the rings were cut 250 μm deep into a sheet of acrylic. Subsequent negatives of pillars were engraved into the rings at a height of 50 μm. The spacing between pillars was optimized to provide a ‘stop’ to maintain the cover slip culture in place over the culture space within the PDMS ring. PDMS base and curing agent was mixed at a ratio of 10:1, degassed for 20 minutes, and cured in the molds for 60 minutes at 80°C. Devices were removed and sterilized in 70% ethanol overnight and stored for up to one month. Coverslips (Chemglass; Vineland, NJ) were acid washed in 1M HCl overnight, followed by 4 washes in ultrapure water for 10 minutes each. Coverslips were soaked in ethanol for one hour and sterilized by UV for 30 minutes.

### Isolation and differentiation of monocyte derived macrophages (MDMs) from peripheral blood mononuclear cells (PBMCs)

Whole blood from healthy females over the age of 18 years was purchased from Innovative Research (Novi, MI). Monocytes were enriched by negative selection using the Rosette Sep^®^ monocyte enrichment cocktail according to manufacturer's instructions (STEMCELL Technologies; Vancouver, Canada). To differentiate isolated monocytes into the M2 phenotype, 9 mm square coverslips were placed in individual wells of a 24 well plate and monocytes were seeded at a density of 200,000 cells/well for 6 days in AIM V media (Invitrogen) supplemented with 1% penicillin-streptomycin in the presence of 20 ng/mL M-CSF (Peprotech; Rocky Hill, NJ). Macrophages were then activated for 48 hours in 2 ng/mL each of IL-4 and IL-13 (Peprotech). Phenotypical characterization of MDM was performed by immunofluorescence after 8 days using anti-CD68 (clone Y1/82A), anti-CD206 (clone 19.2), and their respective isotypes (BD Biosciences; Franklin Lakes, NJ) with a goat anti-mouse Alex Fluor 488 secondary (Invitrogen). Fixed cells were imaged at room temperature in phosphate buffered solution (PBS, Invitrogen) on a Zeiss Axio Observer.Z1 inverted microscope with an AxioCam 506 mono camera, a Plan-NEOFLUOR 20x 0.4-NA air objective, and Zen2 software (Zeiss; Oberkochen, Germany).

### Isolation of patient PBMCs

Informed consent was obtained from recruited blood donors (23 ovarian cancer patients and 21 healthy females) and the study was approved by the Institutional Review Board at the University of Wisconsin-Madison. Blood was collected in heparinized tubes, diluted 1:1 with PBS, gently overlaid on 20 mL Histopaque (GE Healthcare Life Sciences; Pittsburgh, PA), and spun at 1500 rpm for 30 min. PBMCs were collected from the interphase of PBS/Ficoll, transferred to a new tube, diluted with PBS/2% FBS and centrifuged at 1000 rpm for 10 min. The cells were washed twice with PBS/2% FBS, counted and re-suspended in freeze media (90% FBS with 10% DMSO), and stored in liquid nitrogen until further use.

### PBMC RNA extraction and qRT-PCR

Frozen PBMCs were thawed, washed twice with PBS, and RNA was isolated using mirVana miRNA Isolation kit (Ambion, Inc.; Austin, TX). cDNA was generated with 1 μg of RNA using RT^2^ miRNA First Strand Kit (Qiagen; Valencia, CA). Quantitative real time PCR (qRT-PCR) for *HBEGF* was done using pre-validated qPCR primers (Qiagen) and SYBR Green Fluor qPCR Mastermix (Qiagen) in a CFX real time PCR machine (Bio-Rad-Hercules, CA), and each sample was run in triplicate. Fold-change in expression compared to healthy patients was calculated using delta-delta C_T_; mean fold-change is reported.

### Differentiation of THP-1 cell line

THP-1 were differentiated to a macrophage-like phenotype according to previously described protocols [22]. In brief, 9 mm coverslips were placed in individual wells of a 24 well plate and THP-1 were seeded at 150,000 cells/well. THP-1 were treated with 30 ng/mL of PMA for 6 hours followed by 66 hours with 25 ng/mL IL-4 and 25 ng/mL IL-13 (PeproTech).

### Tumor cell-macrophage co-culture

Tissue culture plastic within the PDMS ring was coated with 50 μL of 2% gelatin for 30 minutes. OVCA433 were then seeded into the PDMS rings at a concentration of 2,555 cells/cm^2^ in 40 μL. 24 hours after seeding, cells in the device were washed with 1:1 Medium199:MCDB105 supplemented with 1% penicillin-streptomycin (serum free media, SFM) and serum starved for 24 hours in 40 μL of SFM. Differentiated MDMs and THP-1 were washed with PBS and changed to SFM for 24 hours in preparation for co-culture. Control coverslips for monoculture conditions were prepared by exposing the coverslip to the same differentiation protocol, in order to account for potential non-specific adsorption of differentiation factors. Following serum starvation, macrophage and cell-free control coverslips were inverted and placed on top of the PDMS ring and 40 μL of fresh SFM was added to the culture (Figure 1B). After every 24 hours of culture, 4 μL of SFM was added to the device to counteract evaporation.

To block epidermal growth factor receptor (EGFR), OVCA433 in PDMS rings and macrophages on coverslips were treated with 10 μg/mL of mAb225 [21] for 1 hour prior to co-culture. After coverslips were added to the PDMS rings, fresh SFM supplemented with 10 μg/mL mAb225 was added; control devices were treated with 10 μg/mL mouse IgG1k (Biolegend; San Diego, CA). To block heparin-bound epidermal growth factor (HB-EGF), SFM containing 10 μg/mL of HB-EGF neutralizing polyclonal antibody (R&D Systems; Minnneapolis, MN) was added to the assembled devices; as a control, devices were treated with 10 μg/mL goat polyclonal isotype (R&D Systems). To determine the impact of exogenous HB-EGF, SFM containing 400 pg/mL HB-EGF (Peprotech) was added to the assembled devices. To broadly inhibit MMPs or specifically inhibit MMP-9, OVCA433 in PDMS rings and macrophages on coverslips were pretreated with 10 μM batimastat (Tocris Biosciences; Bristol, United Kingdom) or 5nM MMP-9 inhibitor (Abcam; Cambridge, United Kingdom), respectively, or 0.01% DMSO for 1 hour prior to co-culture. Coverslips were then added to PDMS rings and fresh SFM with 10 μM batimastat, 5 nM MMP-9 inhibitor, or 0.01% DMSO was added to assembled devices. To determine the impact of exogenous MMP-9, 800 pg/mL activated MMP-9 (Millipore; Billerica, MA) was added to assembled devices. To determine the impact of THP-1 conditioned media, conditioned media was collected from THP-1 in monoculture in devices. Devices were retreated after 24 hours with interventions or controls.

### Proliferation assay

Cell proliferation was quantified after 48 hours of co-culture using Click-iT Imaging Assay (Invitrogen). Cell nuclei were counterstained with 5 μg/mL Hoechst 33528 (Invitrogen). Imaging was performed using the equipment described above for MDM differentiation. Four technical replicates per condition were imaged, and for each replicate, four fields of view were imaged in each replicate. EdU+ cells represent those that entered S phase during EdU incubation. Using ImageJ (NIH), all nuclei and EdU-positive nuclei were counted to calculate the percentage of proliferating cells (those that entered S phase).

### EGFR ligand quantification

Macrophage coverslips were added to PDMS rings without OVCA433, treated with 10 μg/mL mAb225 in SFM to prevent binding of EGFR ligands, and retreated after 24 hours. At 48 hours media was collected to measure secreted levels of HB-EGF, transforming growth factor alpha (TGFα), and epidermal growth factor (EGF) by ELISA (R&D Systems). Ligand concentrations were determined from the standard curve using a four-point logistic curve fit. ELISA sensitivities are 31–2000 pg/mL (HB-EGF), 8–500 pg/mL (TGFα) and 3.9–250 pg/mL (EGF).

### MMP quantification

After 48 hours in culture, media from THP-1-only, OVCA433-only, and co-culture devices were collected to measure secreted levels of MMP-2, -7, and -9 by Bioplex assay (Bio-Rad). MMP concentrations were determined from the standard curve using a four-point logistic curve fit. Bioplex sensitivities are 0.819–17,192 ng/mL (MMP-2), 0.026–56 ng/mL (MMP-7), and 0.224–489 ng/mL (MMP-9).

### RNA extraction and qRT-PCR from *in vitro* cultures

RNA was collected using the Micro-RNeasy Extraction kit (Qiagen) and cDNA was synthesized at 60 ng/20 μL using SuperScript III First-Strand Synthesis System (Invitrogen). qRT-PCR was performed on 6 ng of cDNA using primers for *MMP9* and *GAPDH* (Qiagen) and SsoAdvanced Universal SYBR Green Supermix (Bio-Rad), with three samples run in duplicate from each condition.

### *MMP9* siRNA knockdown in co-culture

OVCA433 were seeded at 1,300 cells/cm^2^ and allowed to attach overnight (cell density was adjusted to account for increased culture time in device during siRNA treatment). Coverslips were removed, cells were washed with PBS, and cells were treated for 24 hours with 25 nM ON-TARGETplus *MMP9* or non-targeting pool siRNA (Dharmacon; Lafayette, CO). THP-1 were differentiated as previously stated for 72 hours, washed with PBS, and treated for 24 hours with 50 nM ON-TARGETplus *MMP9* or non-targeting pool siRNA. Each cell type was allowed to recover in SFM for 24 hours prior to use in co-cultures.

### Statistical analysis

All data are presented as mean ± standard deviation. All experiments were repeated at least twice to ensure reproducibility. Proliferation data using primary M2 donor macrophages (*n* = 3 unique donors) were analyzed using either a paired Student's *T*-test or one-way ANOVA followed by Tukey-HSD. THP-1 proliferation assays (*n* = 4 technical replicates per condition), ELISA (*n* = 4 technical replicates per condition), and qRT-PCR (*n* = 3 technical replicates per condition) were analyzed using either Student's *T*-test or one-way ANOVA followed by Tukey-HSD. Tukey-HSD is a statistical analysis method that allows for comparison of all conditions in the dataset while controlling the false-discovery rate [49]. To simplify plots that have multiple comparisons, conditions that are significantly different (*p* < 0.05) are assigned a unique letter. For example, if group 1 and 2 are significantly different, they are assigned ‘a’ and ‘b’, respectively. A label on group 3 of an ‘a’ would indicate it is not different than group 1 but is significantly different than group 2. Adjusted *p*-values for all reported experiments are listed in Supplementary Table S1. All statistical calculations were done in JMP Pro 11 (SAS Institute; Cary, NC).

## SUPPLEMENTARY TABLES AND FIGURES





## Abbreviations

EGF: epidermal growth factor
EGFR: epidermal growth factor receptor
HB-EGF: heparin binding epidermal growth factor
M1: pro-inflammatory macrophage phenotype
M2: anti-inflammatory macrophage phenotype
MDM: monocyte derived macrophage
MMP: matrix metalloproteinase
MMP-1: matrix metalloproteinase 1
MMP-2: matrix metalloproteinase 2
MMP-3: matrix metalloproteinase 3
MMP-7: matrix metalloproteinase 7
MMP-9: matrix metalloproteinase 9
PBMC: peripheral blood mononuclear cell
PDMS: polydimethylsiloxane
SFM: serum-free media
TGFα: transforming growth factor alpha.
