# Supplementary material for: M2 macrophages induce ovarian cancer cell proliferation via a heparin binding epidermal growth factor/matrix metalloproteinase 9 intercellular feedback loop
Source: Oncotarget. 2016 Nov 19;7(52):86608–20. doi: 10.18632/oncotarget.13474 (PMC5349939; doi:10.18632/oncotarget.13474)
Supplement: Supplementary file 1 [file oncotarget-07-86608-s001.pdf]

M2 macrophages induce ovarian cancer cell proliferation via a heparin binding epidermal growth factor/matrix metalloproteinase 9 intercellular feedback loop

Supplementary Materials

Supplementary Table S1: *P*-values for comparisons in Figures 2-7.
See Supplementary_Table_S1

Supplementary Table S2: Expression of EGF ligands in M2 MDMs

	EGF ligand C_t (Avg ± SD)	GAPDH C_t (Avg ± SD)	ΔC_t (Avg ± SD)
HBEGF	36.1 ± 1.0	22.7 ± 0.6	13.5 ± 0.4
EGF	N/D*	22.7 ± 0.6	N/D
TGFA	N/D*	22.3 ± 2.6	N/D

*N/D indicates not detectable after 40 rounds of amplification.

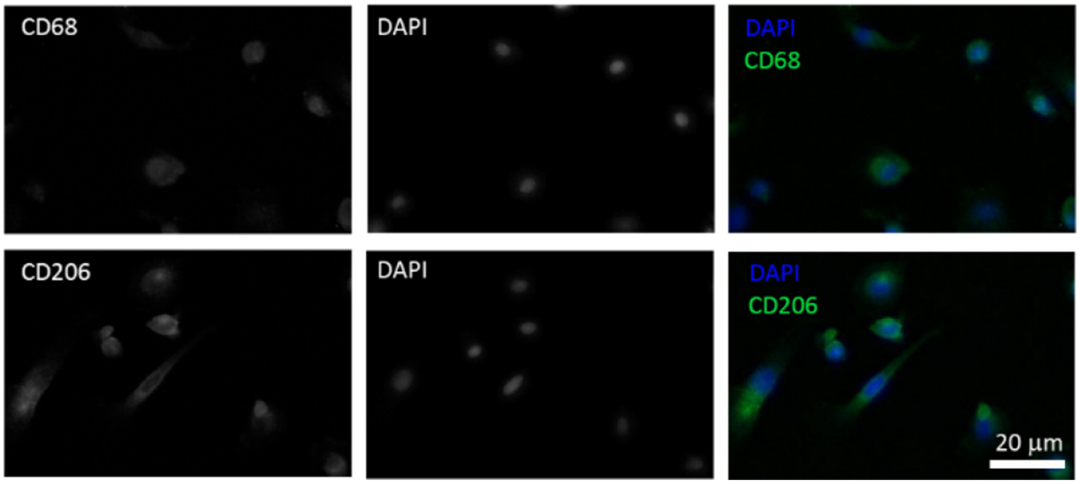

Supplementary Figure S1: CD68 (top) and CD206 (bottom) expression in M2-differentiated MDMs.

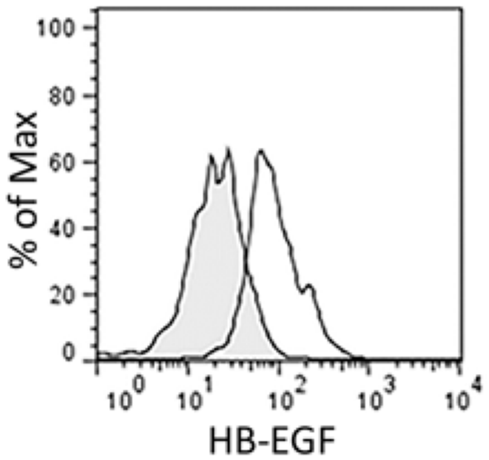

Supplementary Figure S2: Previously frozen stage 3 ovarian cancer patient PBMCs were thawed, washed and recounted for viability. Cells were blocked with donkey IgG (017-000-003, Jackson ImmunoResearch Laboratories) for 15 minutes, then stained with anti-HBEGF primary antibody (AF-259-NA, R&D Systems) for 30 minutes, both on ice. Following these incubations, the cells were washed and incubated with donkey anti-goat NorthernLights 637 secondary antibody (NL002, R&D Systems). After 30 minutes on ice, the cells were washed and stained with CD16-FITC (clone 3G8, 555406) and CD14-PE (clone M5E2, 555398, all from BD Pharmingen). Antibody concentrations used were ≤ 1 μg/million cells. After incubation on ice for 30 minutes, the cells were washed and resuspended in PBS/1% FBS, with propidium iodide at ≤ 1 μg/mL, and samples were collected on a BD FacsCalibur flow cytometer. At least 10,000 live events were collected per sample. The data were analyzed using FlowJo data analysis software. The cells were gated using the following scheme- live events, single events, and then monocyte markers. Monocytes were also distinguished from other cells using size parameters (FSC vs SSC). The expression levels of HB-EGF were then plotted, with an example shown here comparing isotype (grey), and HB-EGF (white) surface expression.

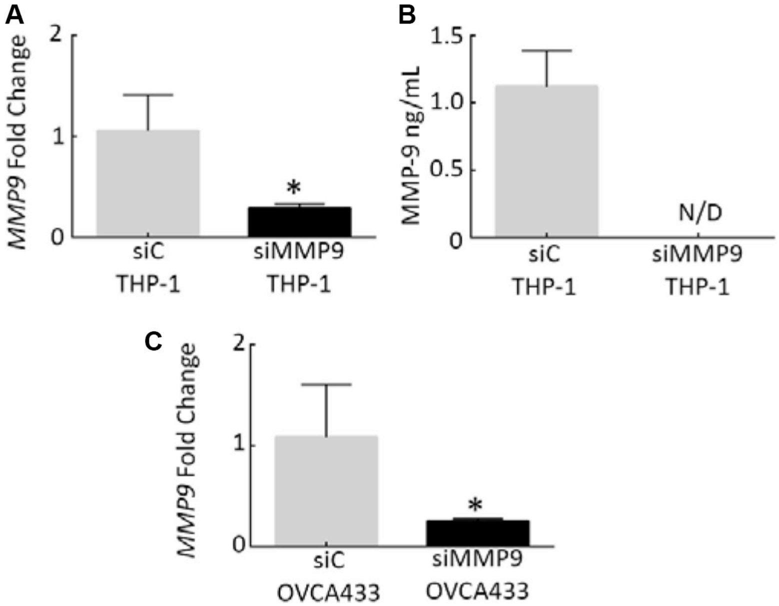

Supplementary Figure S3: (A) *MMP9* expression in M2 THP-1 transfected with 50 nM control siRNA (siC) or *MMP9* siRNA (siMMP9). (**B**) MMP-9 secretion by M2 THP-1 transfected with 50 nM control siRNA (siC) or *MMP9* siRNA (siMMP9). (**C**) MMP9 expression in OVCA433 transfected with 25 nM control siRNA (siC) or *MMP9* siRNA (siMMP9).
