# Supplementary material for: M2 macrophages induce ovarian cancer cell proliferation via a heparin binding epidermal growth factor/matrix metalloproteinase 9 intercellular feedback loop
Source: Oncotarget. 2016 Nov 19;7(52):86608–20. doi: 10.18632/oncotarget.13474 (PMC5349939; doi:10.18632/oncotarget.13474)
Supplement: Supplementary file 2 [file oncotarget-07-86608-s002.docx]

**Supplementary Table S1.** P-values for comparisons in Figures 2-7.

| **Figure** | **Comparison** | **p-value** |
| --- | --- | --- |
| 2B | Monoculture vs CC: Primary Mφ | 0.0147 |
| 2C | Healthy Donors vs Ovca Patients | <0.0001 |
| 2D | Monoculture Isotype vs Monoculture mAb225 | 0.3001 |
|  | Monoculture Isotype vs CC Isotype | 0.0086 |
|  | Monoculture Isotype vs CC mAb225 | 0.5887 |
|  | Monoculture mAb225 vs CC Isotype | 0.0016 |
|  | Monoculture mAb225 vs CC mAb225 | 0.9141 |
|  | CC Isotype vs CC mAb225 | 0.0027 |
| 2E | Monoculture vs CC: THP-1 | 0.0232 |
| 2F | Monoculture Isotype vs Monoculture mAb225 | >0.9999 |
|  | Monoculture Isotype vs CC Isotype | 0.0107 |
|  | Monoculture Isotype vs CC mAb225 | 0.7432 |
|  | Monoculture mAb225 vs CC Isotype | 0.0112 |
|  | Monoculture mAb225 vs CC mAb225 | 0.7653 |
|  | CC Isotype vs CC mAb225 | 0.0311 |
| 3B | Monoculture vs CC: THP-1 | 0.0005 |
|  | Monoculture vs CC + HB-EGF Ab | 0.0746 |
|  | CC: THP-1 vs CC + HB-EGF Ab | 0.0145 |
| 3C | Monoculture vs Monoculture + HB-EGF | 0.0136 |
|  | Monoculture vs CC: THP-1 | 0.0003 |
|  | Monoculture + HB-EGF vs CC: THP-1 | 0.0400 |
| 4A | Monoculture DMSO vs Monoculture Batimastat | 0.1565 |
|  | Monoculture DMSO vs CC DMSO | 0.0145 |
|  | Monoculture DMSO vs CC Batimastat | 0.9785 |
|  | Monoculture Batimastat vs CC DMSO | 0.0003 |
|  | Monoculture Batimastat vs CC Batimastat | 0.2820 |
|  | CC DMSO vs CC Batimastat | 0.0073 |
| 4B MMP-2 | THP-1 vs CC | 0.0823 |
| 4B MMP-7 | THP-1 vs 433 | <0.0001 |
|  | THP-1 vs CC | 0.1176 |
| 4B MMP-9 | THP-1 vs 433 | <0.0001 |
|  | THP-1 vs CC | <0.0001 |
| 5A | Monoculture DMSO vs Monoculture MMP-9 Inh | 0.9778 |
|  | Monoculture DMSO vs CC DMSO | 0.0193 |
|  | Monoculture DMSO vs CC MMP-9 Inh | >0.9999 |
|  | Monoculture MMP-9 Inh vs CC DMSO | 0.0096 |
|  | Monoculture MMP-9 Inh vs CC MMP-9 Inh | 0.9772 |
|  | CC DMSO vs CC MMP-9 Inh | 0.0194 |
| 5B | THP-1 siC vs THP-1 si*MMP9* | 0.0152 |
| 5C | Monoculture vs Monoculture + MMP-9 | 0.9303 |
|  | Monoculture vs CC: THP-1 | <0.0001 |
|  | Monoculture + MMP-9 vs CC: THP-1 | <0.0001 |
| 6A | Monoculture vs Monoculture + CM | 0.1557 |
|  | Monoculture vs CC: THP-1 | 0.0012 |
|  | Monoculture + CM vs CC: THP-1 | 0.0226 |
| 6B | THP-1 Monoculture vs THP-1 Co-culture | >0.9999 |
|  | OVCA433 Monoculture vs OVCA433 Co-culture | 0.0002 |
| 6C | Monoculture Isotype vs Monoculture mAb225 | >0.9999 |
|  | Monoculture Isotype vs CC Isotype | 0.0005 |
|  | Monoculture Isotype vs CC mAb225 | 0.0235 |
|  | Monoculture mAb225 vs CC Isotype | 0.0005 |
|  | Monoculture mAb225 vs CC mAb225 | 0.0224 |
|  | CC Isotype vs CC mAb225 | 0.0427 |
| 7A | OVCA433 siC Monoculture vs OVCA433 si*MMP9* Monoculture | 0.7526 |
|  | OVCA433 siC Monoculture vs OVCA433 siC CC: THP-1 | 0.0004 |
|  | OVCA433 siC Monoculture vs OVCA433 si*MMP9* CC: THP-1 | >0.9999 |
|  | OVCA433 si*MMP9* Monoculture vs OVCA433 siC CC: THP-1 | 0.0010 |
|  | OVCA433 si*MMP9* Monoculture vsOVCA433 si*MMP9* CC: THP-1 | 0.7637 |
|  | OVCA433 siC CC: THP-1 vs OVCA433 si*MMP9* CC: THP-1 | 0.0004 |
| 7B | Monoculture vs THP-1 siC CC: THP-1 | 0.0131 |
|  | Monoculture vs THP-1 si*MMP9* CC:THP-1 | 0.2376 |
|  | THP-1 siC CC: THP-1 vs THP-1 si*MMP9* CC: THP-1 | 0.0011 |
| 7C | OVCA433 siC Monoculture vs OVCA433 si*MMP9* Monoculture | 0.6675 |
|  | OVCA433 siC Monoculture vs OVCA433 siC CC: Primary Mφ | 0.0036 |
|  | OVCA433 siC Monoculture vs OVCA433 si*MMP9* CC: Primary Mφ | 0.9983 |
|  | OVCA433 si*MMP9* Monoculture vs OVCA433 siC CC: Primary Mφ | 0.0010 |
|  | OVCA433 si*MMP9* Monoculture vsOVCA433 si*MMP9* CC: Primary Mφ | 0.7578 |
|  | OVCA433 siC CC: Primary Mφ vs OVCA433 si*MMP9* CC: Primary Mφ | 0.0029 |
